# Expression of RNA-Interference/Antisense Transgenes by the Cognate Promoters of Target Genes Is a Better Gene-Silencing Strategy to Study Gene Functions in Rice

**DOI:** 10.1371/journal.pone.0017444

**Published:** 2011-03-03

**Authors:** Jing Li, Dagang Jiang, Hai Zhou, Feng Li, Jiawei Yang, Laifa Hong, Xiao Fu, Zhibin Li, Zhenlan Liu, Jianming Li, Chuxiong Zhuang

**Affiliations:** 1 Key Laboratory of Plant Functional Genomics and Biotechnology, Education Department of Guangdong Province, South China Agricultural University, Guangzhou, People's Republic of China; 2 Department of Molecular, Cellular, and Developmental Biology, University of Michigan, Ann Arbor, Michigan, United States of America; King's College London, United Kingdom

## Abstract

Antisense and RNA interference (RNAi)-mediated gene silencing systems are powerful reverse genetic methods for studying gene function. Most RNAi and antisense experiments used constitutive promoters to drive the expression of RNAi/antisense transgenes; however, several reports showed that constitutive promoters were not expressed in all cell types in cereal plants, suggesting that the constitutive promoter systems are not effective for silencing gene expression in certain tissues/organs. To develop an alternative method that complements the constitutive promoter systems, we constructed RNAi and/or antisense transgenes for four rice genes using a constitutive promoter or a cognate promoter of a selected rice target gene and generated many independent transgenic lines. Genetic, molecular, and phenotypic analyses of these RNAi/antisense transgenic rice plants, in comparison to previously-reported transgenic lines that silenced similar genes, revealed that expression of the cognate promoter-driven RNAi/antisense transgenes resulted in novel growth/developmental defects that were not observed in transgenic lines expressing constitutive promoter-driven gene-silencing transgenes of the same target genes. Our results strongly suggested that expression of RNAi/antisense transgenes by cognate promoters of target genes is a better gene-silencing approach to discovery gene function in rice.

## Introduction

Plant genomic research has made remarkable progress in recent years. The genome sequence of a plant provides the foundation for detailed functional characterization of plant genes [1]. Rice was the first crop plant to have its complete genome sequenced [2]. Although 56,797 genes have been annotated from sequencing of the rice genome [3], [4], the functions of >60% of these predicted genes are unknown. Therefore, one of the most challenging goals of the rice functional genomics is to characterize the functions of these unknown rice genes.

Reverse genetics is a powerful tool for assessing gene function [5], and several reverse genetics approaches have been developed in recent years for functional genomic studies. Transfer DNA (T-DNA) insertional mutagenesis that creates loss of function mutations [6] is a very effective reverse genetics approach in studying gene functions. Although T-DNA insertional mutagen has been widely used, it has several disadvantages. One common drawback is complex organizations of many T-DNA inserts, resulting in an overall 40% to 50% failure rate in identifying the exact T-DNA insertional site [7]. Besides, T-DNA exhibits certain integration preference and may therefore not saturate the entire rice genome [8]. As a result, only 27,551 rice genes were found to be mutated by T-DNA insertions from collections of >400,000 independent rice T-DNA lines [8]. In addition, T-DNA insertion may lead to lethal phenotypes, preventing genetic studies of gene functions, or cause no observable phenotype due to functional redundancy of homologous genes.

Several alternative reverse genetic approaches to study gene function, such as RNA interference (RNAi) and antisense RNA technology could circumvent the limitations of T-DNA insertional mutagenesis. In RNAi technology, the introduction of double-stranded RNAs (dsRNAs) into cells inhibits the expression of the corresponding endogenous gene at transcriptional and post-transcriptional levels [9]. RNAi could silence the expression of an endogenous target gene without altering its gene structure or producing the permanent loss of gene function. The partial gene silencing-effect of the RNAi and antisense strategies could avoid potential lethality of a T-DNA insertional mutation. In addition, RNAi/antisense-initiated gene silencing could simultaneously inhibit the expression of several homologous genes, thus overcoming potential gene redundancy problems. These advantages have made the RNAi and antisense RNA strategies the method of choice for studying gene functions in plants in recent years.

The choice of promoter is a very important factor in RNAi and antisense RNA strategies. The most commonly used promoters in RNAi and antisense strategies are constitutive promoters, such as the *35S* promoter from cauliflower mosaic virus (*pCaMV35S*) [10] and the promoter from the maize *Ubiquitin-1* gene (*pUbi1*) [11]. Without species restriction, constitutive promoters drive high expression in virtually all tissues/organs of transgenic plants independently of tissue/organ-specific regulators or developmental/environmental signals. However, the constitutive promoter-driven expression of an RNAi/antisense-transgene could cause pleiotropic phenotypes or embryo lethality by silencing the expression of the target gene and its homologs, thus making it extremely difficult to study the functions of the target gene or to define a causal relationship between a silenced gene and the observed phenotypic alterations. On the other hand, recent studies revealed that constitutive promoters are not active in all cell types, especially in cereal crops [12], [13]. Therefore, gene functions cannot be fully defined, as the expression pattern of an RNAi/antisense transgene might not completely overlap with that of its target gene.

Regulated promoters such as organ/tissue- or developmental stage-specific promoters [14], [15] and physically/chemically-inducible promoters [16], [17], [18], [19], [20] have been used in the past to better control the expression of an RNAi/antisense transgene avoiding the adverse effects of constitutive promoters. However, these promoters have their own limitations as an RNAi/antisense-transgene driven by a regulated promoter will only be expressed in certain tissues/organs, at specific developmental stages, or in response to a unique chemical/physical signal but has no effect on the target gene in other relevant tissues/organs at certain important developmental stages [21].

By contrast, a cognate promoter of a target gene should drive the expression of a gene-knockdown RNAi/antisense-transgene in the native expression domains of the targeted endogenous gene, which could overcome many of the known limitations of constitutive/regulated promoters in driving the expression of gene-silencing transgenes to define the biological functions of their corresponding endogenous genes.

In this study, we investigated the effectiveness of constitutive/cognate promoter-driven RNAi/antisense-transgene in causing growth/developmental phenotype in transgenic rice plants. Four rice genes, *Pyruvate Dehydrogenase Kinase 1* and 2 (*OsPDK1* and *OsPDK2*), *Silencing Information Regulator 2* (*OsSRT1*), and *Metallothionein2b* (*OsMT2b*), were selected for our studies. The physiological functions of these four genes were previously studied by gene silencing using constitutive promoter-driven RNAi/antisense transgenes [22], [23], [24], however, our studies using the cognate promoter-driven RNAi/antisense transgenes revealed additional functions of these genes in regulating rice growth/development. Our investigation with the two *OsPDK* genes also showed that the cognate promoter approach could specifically reduce the transcript level of one member gene without affecting the expression of other members of a gene family.

## Results

### The cognate promoter-driven expression of an RNAi-transgene revealed novel physiological functions of OsMT2b

Metallothioneins (MTs) are a family of low-molecular weight, cysteine rich intracellular proteins that are thought to play important roles in metal tolerance, detoxification, and homeostasis in plants via binding heavy metals [22], [25], [26]. The rice genome encodes 15 MT proteins that could be classified into four types [22]. OsMT2b, a type-2 MT, scavenges reactive oxygen species [22], [27]. Earlier studies using transgenic rice plants in which *OsMT2b* was silenced by an *OsMT2b*-RNAi transgene driven by the maize *Ubi* promoter showed that OsMT2b participates in epidermal cell death [28] and is involved in root development and seed embryo germination by modulating the endogenous cytokinin level [22].

**Figure 1:**
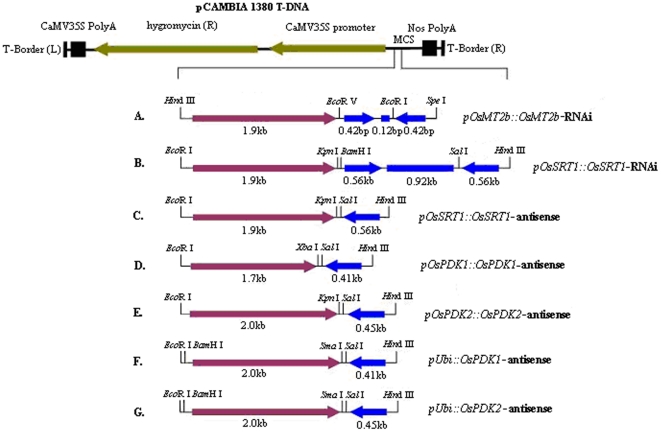
Schematic presentation of the constructed RNAi/antisense transgenes. (A, B) Positions and orientations of independently amplified genomic/cDNA fragments for generating *pOsMT2b::OsMT2b* and *pOsSRT1::OsSRT1* RNAi transgenes. (C–E) Schematic presentation of antisense transgenes of *OsSRT1* (C), *OsPDK1* (D) and *OsPDK2* (E) driven by their cognate promoters. (F, G) Schematic presentation of the *pUbi::OsPDK1* (F) and *pUbi::OsPDK2* (G) antisense transgenes. Purple arrows represent promoters, blue right arrows indicate sense fragments, blue left arrows mean antisense fragments, and blue bars denote introns.

**Figure 2:**
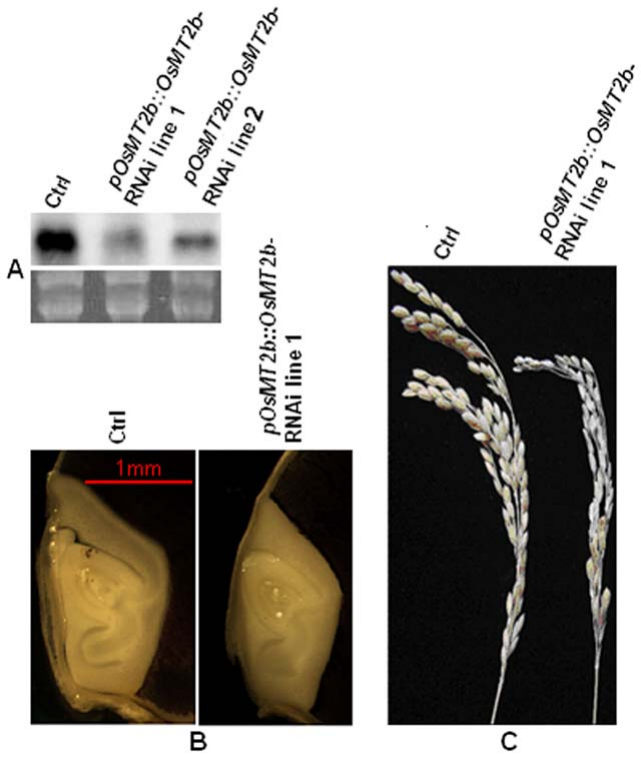
Phenotypic and RNA blot analyses of primary *OsMT2b*-RNAi transgenic lines. A. RNA blot analysis of the endogenous *OsMT2b* transcript. Twenty µg of total RNAs isolated from two independent *pOsMT2b::OsMT2b*-RNAi transgenic lines (1 and 2) and the control line (Ctrl) were separated on denaturing agarose gel, stained with ethidium bromide, transferred to a nylon membrane, hybridized with α ^32^P-labeled *OsMT2b* cDNA fragment, and analyzed by autoradiography (upper panel). The lower panel shows the ethidium bromide-stained ribosomal RNAs used as a loading control. B. Comparison of the seed embryo between a representative *OsMT2b*-RNAi transgenic line (1) and the control (Ctrl). Scale Bar = 1 mm. C. Phenotypic comparison of panicles/spikelets between the representative *pOsMT2b::OsMT2b*-RNAi transgenic line (1) and the control (Ctrl).

To better understand the physiological functions of OsMT2b, we generated an *OsMT2b* RNAi transgene driven by the cognate promoter of the endogenous *OsMT2b* gene (Figure 1A) and transformed the resulting *pOsMT2b::OsMT2b*-RNAi transgene into wild-type rice plants. Ten independent transgenic lines were obtained and carefully analyzed, among which 6 transgenic lines exhibited phenotypic variations in the T_0_ generation. RNA blot analyses found that the expression of the endogenous *OsMT2b* gene was significantly reduced in two independent *pOsMT2b::OsMT2b*-RNAi transgenic lines exhibiting the growth defects (Figure 2A), while segregation analysis of T_1_ progeny of several T_0_ lines carrying single-copy transgene revealed a 3∶1 ratio for normal individuals vs. abnormal individuals. Analyses of the morphological/developmental defects of the 6 independent T_0_ transgenic lines and their offspring not only confirmed previously reported phenotypic alterations, including smaller mature embryos and reduced thickness of scutellum of embryos (Figure 2B), but also discovered novel growth phenotypes such as smaller spikelets, lower percentage of seed setting, and smaller seeds at the bottom of spikes (Figure 2C). Our study thus revealed a functional role of OsMT2b in spikelet/seed development, suggesting that the cognate promoter-driven gene silencing is a better strategy than the constitutive promoter-driven gene silencing to study gene functions in rice.

### Silencing of the rice *OsSRT1* gene by cognate promoter-driven *OsSRT1*-RNAi or *OsSRT1*-antisense transgenes

To further confirm our discovery, we generated a cognate promoter-driven RNAi transgene for another rice gene, which encodes a protein homologous to the SILENT INFORMATION REGULATOR2 (SIR2), a highly conserved NAD^+^-dependent protein deacetylase [29], [30]. The rice genome encodes two SIR2-related proteins, named OsSRT1 and OsSRT2 [23], [31]. An earlier study showed that transgenic rice plants in which *OsSIRT1* was silenced by an *OsSRT1*-RNAi transgene driven by the *CaMV35S* promoter exhibited brown dots on leaves, which became larger at later stages, leading to premature leaf senescence [23].

**Figure 3:**
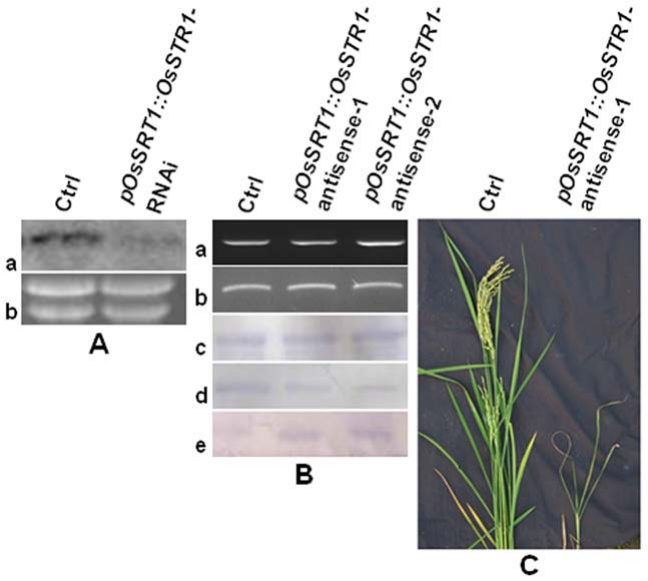
Phenotypic, molecular, and biochemical analyses of primary *OsSRT1* antisense transgenic lines. A. RNA blot analysis of the *OsSRT1* transcript. Twenty µg of total RNAs isolated from calli derived from the control rice plant (Ctrl) and hygromycin-resistant calli transformed with the *pOsSRT1::OsSRT1*-RNAi transgene were separated on denaturing agarose gel, stained with ethidium bromide, transferred to a nylon membrane, hybridized with α-^32^P-labeled *OsSRT1* cDNA fragment, and analyzed by autoradiography(a). The lower panel shows the ethidium bromide-stained ribosomal RNAs used as a loading control (b). B. The expression of the *pOsSRT1::OsSRT1*-antisense transgene had no effect on the *OsSRT1* mRNA level but significantly reduced the OsSRT1 protein abundance. *pOsSRT1::OsSRT1*-antisense-1 and -2 are two independent *OsSRT1*-antisense transgenic lines. a) RT-PCR analysis of the transcript abundance of the endogenous *OsSRT1* gene (see Materials and Methods for experimental details). b) *β-actin* was used as a loading control. c–e) Immunoblot analysis of the protein abundance of Tubulin (c), OsSRT1(d), and the level of H3K9 acetylation(e). Equal amounts of protein crude extracts were separated by SDS-polyacrylamide gel electrophoresis, transferred to nitrocellulose filters, and analyzed by immunoblotting with antibodies against Tubulin (for loading control), OsSRT1, and acetylated Lys-9 residue of histone 3 (H3K9). C. Phenotypic comparison between a representative *pOsSRT1::OsSRT1* antisense transgenic line (1) and a wild-type control (Ctrl).

Despite numerous attempts, we were unable to generate a single *pOsSRT1::OsSRT1*-RNAi (Figure 1B) transgenic rice line from the *OsSRT1*-RNAi transgene-transformed calli. We suspected that the RNAi-mediated silencing of *OsSRT1* in its native expression domains prevented transformed calli to regenerate. To test our hypothesis, we performed a Southern blot analysis with genomic DNAs isolated from antibiotic-resistant calli and found that these hygromycin-resistant calli carried the hygromycin-B-phosphotransferase gene, the antibiotic marker gene of the *pOsSRT1::OsSRT1*-RNAi plasmid and originated from different transformation events (data not shown). We also performed RNA blot analysis using total RNAs isolated from hygromycin-resistant and control calli and found that the *OsSRT1* transcript level was significantly reduced in hygromycin-resistant calli (Figure 3A). Given the successful generation of multiple transgenic lines when an *OsSRT1*-RNAi transgene was driven by the *CaMV35S* promoter [23], our use of a cognate promoter-driven RNAi-transgene revealed a novel role of *OsSRT1* in tissue regeneration.

Because no transgenic plants were obtained with the *pOsSRT1::OsSTR1*-RNAi transgene, we created a *pOsSRT1::OsSRT1* antisense transgene carrying the cognate promoter of the endogenous *OsSRT1* gene (Figure 1C), as an antisense transgene is less effective in triggering gene silencing. A total of 12 independent transgenic lines were produced but none of them exhibited any observable growth alteration. However, at least 5 T_0_ lines segregated out T_1_ individuals displaying developmental defects with a 3∶1 ratio of normal plants vs. defective individuals (data not shown). Further genetic studies suggested that the defective T_1_ plants are likely homozygous for the *pOsSRT1::OsSRT1*-antisense transgene as they failed to segregate out normal plants in 4 subsequent generations. Two homozygous *pOsSRT1::OsSRT1*-antisense lines were selected to determine the gene silencing effect of the cognate-promoter-driven antisense transgene.

Although RT-PCR analysis detected no significant changes in the *OsSRT1* transcript level (Figure 3B-a), our immunoblot experiment showed that the OsSRT1 protein abundance in the two *pOsSRT1::OsSIRT1*-antisense transgenic lines was significantly reduced (Figure 3B-d). Consistent with the known function of the yeast/mammalian SIR2 proteins in deacetylating the acetylated lysine-9 residue on histone 3 (H3K9) [23], an immunoblot assay using antibodies raised against the acetylated H3K9 revealed the increased H3K9 acetylation in the two selected transgenic lines (Figure 3B-e) , further supporting a reduction of OsSRT1 abundance in the two selected transgenic lines. These homozygous *pOsSRT1::OsSRT1*-antisense transgenic rice plants not only displayed brown spots on the leaves and early senescence symptom (Figure 3C), which are similar to what were previously observed on *pCaMV35S::OsSRT1*-RNAi transgenic plants [23], but also exhibited additional growth/developmental abnormalities, such as decreased tillering capacity and lower seed setting (Figure 3C and data not shown). Our studies using *pOsSRT1::OsSRT1*-RNAi/antisense transgenes therefore further supported our conclusion that expression of RNAi/antisense transgene using a cognate promoter of the target gene is a better silencing strategy in revealing its physiological functions in rice.

### Direct comparison of the phenotypic differences of constitutive and cognate promoters in driving the expression of antisense transgenes in rice

To directly compare the differential effects of constitutive and cognate promoters on silencing rice genes, we created two antisense transgenes each for two highly-homologous rice genes encoding pyruvate dehydrogenase kinase 1 and 2 (OsPDK1 and OsPDK2), one using the maize *Ubi* promoter and the other with the cognate promoters of the *OsPDK* genes (Figure 1D–1G). An earlier study showed that silencing the *OsPDK1* gene by a *CaMV35S* promoter-driven *OsPDK1*-RNAi transgene resulted in a weak dwarf phenotype in transgenic rice plants [24].

**Figure 4:**
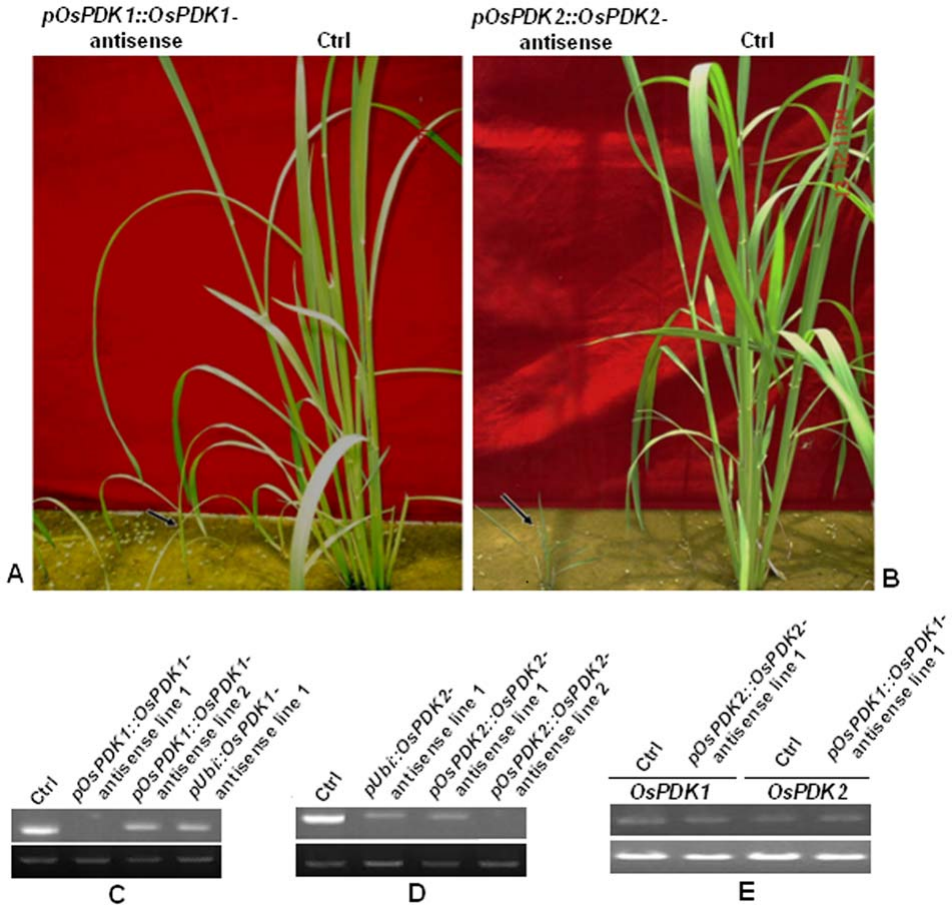
Similar and different growth/developmental defects in *pOsPDK::OsPDK*-antisense transgenic plants. A. Phenotypic comparison between a representative *pOsPDK1::OsPDK1*-antisense transgenic line (indicated by black arrow) and the wild-type control (Ctrl) of the same developmental age (booting stage). B. Phenotypic comparison between a representative *pOsPDK2::OsPDK2*-antisense transgenic line (indicated by black arrow) and the wild-type control (Ctrl) of the same developmental age (booting stage). C–E. RT-PCR analysis of the transcript abundance of the endogenous *OsPDK1* and *OsPDK2* genes in various *pUbi/pOsPDK::OsPDK*-antisense transgenic plants. Equal amounts of total RNAs isolated from the wild-type control (Ctrl) and selected transgenic plants were converted into 1^st^ cDNAs. Half microliter of the resulting 1st-strand cDNAs was used as templates for PCR-amplification using gene-specific primers (see Materials and Methods for details) of the transcripts of the endogenous *OsPDK1* (C plus lanes 1 and 2 in E) and *OsPDK2* (D plus lanes 3 and 4 in E) genes. RT-PCR analysis of the rice *β-actin* gene (the lower strip in each panel) was used as a control.

Transformation of *pOsPDK1::OsPDK1* and *pOsPDK2::OsPDK2* antisense transgenes resulted in generation of 16 and 13 independent transgenic lines of Zhonghua 11, respectively. Both transgenes caused two types of growth alterations.The first one is slightly-reduced plant height (∼10% reduction compared to the control), resembling that of the previously-reported *pUbi::OsPDK1*-RNAi transgenic lines [24]. The other type of growth defects included severe dwarfism (∼90% shorter than the control), single tillering, and semi-sterility (Figure 4A and 4B), which were not observed in *p35S::OsPDK1*-RNAi transgenic rice plants. RT-PCR analysis revealed a slight reduction of the *OsPDK* transcript abundance in weakly dwarfed transgenic plants but detected no *OsPDK* transcripts in severely dwarfed lines (Figure 4C and 4D). Interestingly, despite high sequence similarity between the two *OsPDK* genes, the antisense-triggered gene silencing was quite specific as the transcript levels of *OsPDK1* and *OsPDK2* were not obviously changed in *OsPDK2*-antisense and *OsPDK1*-antisense transgenic plants, respectively (Figure 4E). Consistently, the severely-dwarfed *pOsPDK1::OsPDK1* and *pOsPDK2::OsPDK2*-antisense transgenic lines also exhibited unique phenotypes. The *pOsPDK1::OsPDK1*-antisense lines had longer life cycle than the control plants with pale yellow leaves, whereas *pOsPDK2::OsPDK2*-antisense dwarfs had shorter life cycle than the control with darker green leaves (Figure 4A and 4B), revealing different physiological functions for two highly homologous rice proteins.

**Figure 5:**
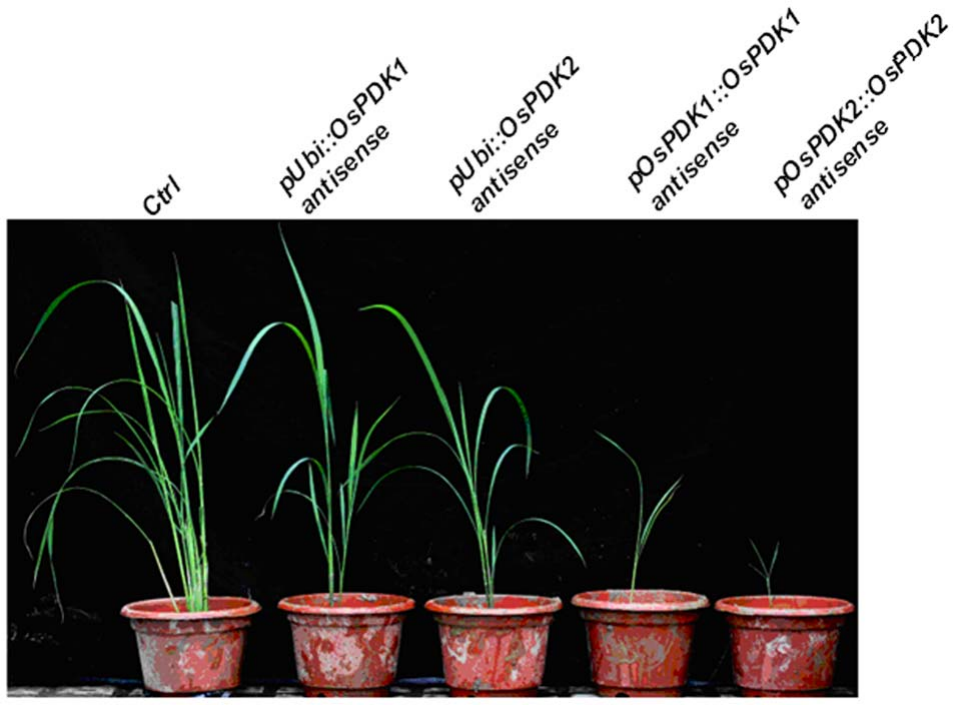
Phenotypic comparison between *pOsPDK::OsPDK*-antisense and *pUbi::OsPDK*-antisense transgenic plants. Shown here from left to right are six-week old soil-grown rice plants of the wild-type control (Ctrl) and a representative transgenic line carrying an antisense transgene of *pUbi::OsPDK1*, *pUbi::OsPDK2*, *pOsPDK1::OsPDK1* and *pOsPDK2::OsPDK2*.

By contrast, expression of either *OsPDK*-antisense transgene driven by the constitutively-active *Ubi* promoter failed to cause extreme dwarfism but only resulted in the semidwarf phenotype (∼30% shorter than control plants) (Figure 5), which is slightly stronger than that caused by the expression of *pUbi::OsPDK1*-RNAi transgene [24]. Consistently, RT-PCR analysis revealed a slight reduction of *OsPDK1* or *OsPDK2* transcript level in the *pUbi::OsPDK*-antisense transgenic lines (Figure 4C and 4D). As expected from the failure of the *pUbi::OsPDK*-antisense transgenes to cause strong dwarfism, no obvious phenotypic difference was observed between *pUbi:OsPDK1*- and *pUbi:OsPDK2*-antisense transgenic plants. Taken together, our direct comparison study clearly demonstrated the superiority of the cognate promoter-driven transgenes in silencing the corresponding endogenous rice genes to reveal their physiological functions.

## Discussion

In this study, we investigated the differential effects of constitutive promoter-driven and cognate promoter-driven RNAi/antisense transgenes on gene silencing and causing growth/developmental defects in transgenic rice plants. By comparing the growth/developmental phenotypes of our transgenic plants with those of previously reported transgenic lines, we found that the expression of the cognate promoter-driven RNAi/antisense transgenes often gave rise to growth/developmental defects that were not observed on transgenic lines expressing constitutive promoter-driven RNAi/antisense transgenes of the same target genes. For example, some *pOsPDK1::OsPDK1*-antisense transgenic lines were severe dwarfs with yellow leaves, which were not observed in previously reported *pUbi/p35S::OsPDK1*- antisense transgenic rice plants [24]. On the other hand, the use of a cognate promoter-driven RNAi/antisense transgene could avoid potential lethal phenotype caused by expression of a constitutive promoter-driven RNAi/antisense transgene. For example, an earlier study reported that strong silencing of the *OsSRT1* gene caused a postembryonic lethal phenotype in *p35S::OsSRT1*-RNAi transgenic plants [23], whereas no such a phenotype was observed in our *pOsSRT1::OsSRT1*-antisense transgenic lines. Our results demonstrated that gene silencing using cognate promoter-driven RNAi/antisense transgenes was a more effective and physiologically relevant approach than that driven by constitutive promoters to define the gene functions in rice. We have so far adopted this approach to investigate the physiological functions of more than 20 rice genes (unpublished data).

Antisense RNA, with the formation of antisense/target dsRNA, is a gene silencing mechanism resulting in mRNA degradation or affecting mRNA translation [32], [33]. It has been reported that the binding position of the antisense RNA may determine gene-silencing mechanisms [34], [35]. Antisense RNAs binding to the 3′ untranslated region (3′-UTR) represses translation [32], whereas antisense RNAs pairing with the 5′ UTR of the target gene could cause mRNA degradation. The full-length of *OsSRT1* (NM_001058878) cDNA is 1891 bp, and the predicted antisense transcript of the *OsSRT1*-antisense transgene would hybridize to the region near the 3′-end, between nucleotides 1206 and 1770, of the endogenous *OsSRT1* transcript. In *pOsSRT1::OsSRT1*-antisense transgenic plants, the transcript level of the endogenous *OsSRT1* gene was not obviously changed but the OsSRT1 protein level and its predicted histone deacetylase activity were significantly reduced. The same antisense-transgene construction strategy was used for 8 additional rice genes, and similar effects on the abundance of the endogenous target genes and their protein products were observed (data not shown). The full-length of *OsPDK1* (NM_001056731.1) and *OsPDK2* (NM_001066936.1) cDNAs are 1535 bp and1480 bp, respectively. The cDNA fragments used for making antisense transgenes of *OsPDK1* and *OsPDK2* were derived from the regions spanning 434–845 bp and 153–594 bp near the 5′ ends of *OsPDK1* and *OsPDK2* cDNAs, respectively. In *pOsPDK1::OsPDK1* and *pOsPDK2::OsPDK2*-antisense transgenic progeny, the mRNA levels of the corresponding target genes decreased significantly. Our studies thus further supported an earlier hypothesis that antisense RNA directed against the 5′ UTR often results in degradation of the target mRNA whereas antisense RNA targeted near the 3′ UTR causes translational inhibition.

Consistent with earlier discoveries that the gene-silencing efficiency of antisense transgene is lower than that of RNAi-transgene, growth/developmental defects were only observed in the homozygous progeny of *OsSRT1*/*OsPDK1*/*OsPDK2*-antisense transgenic lines. However, such a lower gene-silencing efficiency could be useful to avoid lethal phenotypes of RNAi-induced strong gene silencing. For example, despite numerous attempts and successful generation of antibiotic-resistant calli with an *pOsSRT1::OsSRT1*-RNAi transgene, no single *pOsSRT1::OsSRT1*-RNAi transgenic plants was regenerated; however, we were quite successful in generating *pOsSRT1::OsSRT1*-antisense transgenic lines with reduced transcript level of the endogenous *OsSRT1* gene. We suggest that the antisense-mediated gene-silencing technology might be more appropriate than the RNAi technology to study rice genes that play roles in early stage of plant growth/development.

Gene redundancy is a major obstacle in functional genomic studies. 53% and 68% of the non-transposable element-related genes in rice and *Arabidopsis* are grouped into paralogous gene families, respectively [36]. Although family members show high sequence homology at the nucleic acid level, they often have different expression patterns and biological functions. Gene-silencing using a constitutive promoter-drive RNAi/antisense transgene could simultaneously knockdown the intended target gene and its potential homologs [37], making is extremely difficult in assigning a given biological function to a member of that gene family. For example, a recent report showed that four members of *OsAGO1* gene family, *OsAGO1a*, *OsAGO1b*, *OsAGO1c*, and *OsAGO1d*, are highly similar in sequence with each other [38], and their transcript levels were all significantly reduced by the expression of a constitutive promoter-driven *OsAGO1-RNAi* transgene. In this work, we studied two members of the *OsPDK* gene family, *OsPDK1* and *OsPDK2*, which share 82% similarity at the nucleotide level. *OsPDK1* is expressed in mature leaves, while *OsPDK2* is mainly expressed in actively-growing tissues. As expected, no phenotypic difference was observed in *pUbi::OsPDK1*/*pUbi::OsPDK2*-antisense transgenic lines, making it difficult to define the physiological function for each *OsPDK* gene. However, transgenic plants in which the *OsPDK1*/*OsPDK2*-antisense transgene was driven by the corresponding cognate promoter displayed different phenotypes. The *pOsPDK1::OsPDK1*-antisense transgene caused yellowish leaf color and longer life cycle, whereas the expression of the *pOsPDK2::OsPDK2*-antisense transgene resulted in darker green leaf color and a shortened life cycle with precocious flowering. Our results thus suggested that the expression of an antisense transgene by the cognate promoter of its target gene might be a better strategy to study the physiological functions of gene families.

## Materials and Methods

### Plant and Other Experimental Materials

Rice (*Oryza sativa* L. ssp. *Japonica*) cv. Zhonghua 11 was used for all experiments. Transgenic plants were grown in a greenhouse with normal daylight illumination. *Escherichia coli* DH10B and *Agrobacterium tumefaciens* strain EHA105 were used for cloning and transformation experiments. pCAMBIA1380 was used as the binary vector for *Agrobacterium*-mediated transformation [39].

### Plasmid Construction

Two RNAi transgenes (*OsSRT1* and *OsMT2b*) and three antisense transgenes (*OsSRT1*, *OsPDK1* and *OsPDK2*) were constructed (Text S1). These 5 transgenes were driven by the cognate promoters of the corresponding target genes. To directly investigate the differential effect of cognate promoters and constitutive promoters on gene silencing, *OsPDK1* and *OsPDK2* antisense transgenes driven by the maize *pUbi* promoter were also constructed. Primers were designed based on published cDNA sequences of *OsSRT1*, *OsMT2b*, *OsPDK1* and *OsPDK2* (Table 1) and were used to amplify gene-specific cDNA fragments from total RNAs isolated from Zhonghua 11. The published genome sequences were also used to locate the 2.0-kb genomic fragment immediately upstream of the annotated ATG start codon for each gene (Table 2), which were amplified by PCR using the primer pairs listed in Table 1 and used as cognate promoters for RNAi/antisense transgene construction. The intron fragments of RNAi transgenes were directly amplified the genomic DNA of Zhonghua 11 (Figure 1A and 1B). Each of the constructed transgenes was fully sequenced to ensure no PCR error before being transformed into *Agrobacterial* cells.

**Table 1:** Sequences of primers.

Names of primers	abbreviation	sequence (5′to 3′)	Description
*OsMT2b* promoter f	P-MT-F	aaaaaagcttgagatgctaatcaagtctctctg	*Hin*d III
*OsMT2b* promoter r	P-MT-R	aaaagatatcagatgttgttgctgattgagctc	*Eco*R V
*OsSRT1* promoter f	P-SRT-F	aaaagaattcgtgcttgtgtgtcattctaccc	*Eco*R I
*OsSRT1* promoter r	P-SRT-R	aaaaggtaccggacatggtggttcagttgaaccc	*Kpn* I
*OsPDK1* promoter f	P-PDK1-F	aaaagaattcgtagtgtcaggctgtcagcaac	*Eco*R I
*OsPDK1* promoter r	P-PDK1-R	aaaatctagaccctaccgacaacagcaccac	*Xba* I
*OsPDK2* promoter f	P-PDK2-F	aaaagaattccgctgtactatgagtcgtacc	*Eco*R I
*OsPDK2* promoter r	P-PDK2-R	aaaaggtaccatcatgtagcgcaggctcac	*Kpn* I
*Ubi* promoter f	P-Ubi-F	aaaaggatccagtgcagcgtgacccggtc	*Bam*H I
*Ubi* promoter r	P-Ubi-R	aaaacccgggcagaagtaacaccaaacaacagg	*Sma* I
*OsMT2b* RNAi 1	R-MT-1	aaaagaattcgctgctccatccaacaagg	*Eco*R I
*OsMT2b* RNAi 2	R-MT-2	aaaagatatcgaagcctggcacgcatgagg	*Eco*R V
*OsMT2b* RNAi 3	R-MT-3	aaaaactagtgaagcctggcacgcatgagg	*Spe* I
*OsSRT1* RNAi 1	R-SRT-1	aaaagtcgacggctgttcgagctcttccattg	*Sal* I
*OsSRT1* RNAi 2	R-SRT-2	aaaaggatccataccatcaagccccacaaccag	*Bam*H I
*OsSRT1* RNAi 3	R-SRT-3	aaaaaagcttcataccatcaagccccacaaccag	*Hin*d III
*OsPDK1* sense f	S-PDK1-F.	aaaagtcgactgggtctccatatatgttcac	*Sal* I
*OsPDK1* sense r	S-PDK1-R	aaaaaagcttggactcattccgcgacttac	*Hin*d III
*OsPDK2* sense f	S-PDK2-R	aaaagtcgacgccaggctctgggtcag	*Sal* I
*OsPDK1* sense r	S-PDK2-R	aaaaaagcttcgggtcgcgccccacg	*Hind* III

**Table 2:** Promoter locations.

Promoters	BAC clones	Locations in the BAC	Gene names	Locations in the rice genome
*OsMT2b*	AC079356	90035∼91973	Os05g0111300	−49∼-1987
*OsSRT1*	AL663014	143857∼141928	Os04g0271000	−49∼-1978
*OsPDK1*	AC082644	112200∼113877	Os03g0370000	−24∼-1701
*OsPDK2*	AP003749	87838∼85738	Os07g0637300	+94∼-2006

Note: “+” means upstream of ATG and “−” means downstream of ATG.

### Plant transformation

To investigate the effectiveness of generated RNAi/antisense transgenes in silencing their target genes, these transgenes was then transformed into the *A. tumefaciens* strain EHA105, which were used to transform rice calli generated from mature dry seeds of Zhonghua11 following a previously described protocol [39]. Tranformed calli were allowed to generate T_0_ plants. After further analyses, they were transferred into soil to produce T_1_ seeds for the generation of T_1_ transgenic lines.

### RNA preparation

Total RNAs were extracted using the Trizol method (Invitrogen) according to the manufacturer's protocols. Briefly, 0.1 g plant tissues from leaves and spikelets of different developmental stages of control/transgenic rice plants were ground in liquid N_2_ to fine powder, dissolved in the Trizol reagent, incubated at 15–30°C for 5 min, mixed with chloroform (0.2 mL/1 mL Trizol reagent), and centrifuged 12,000× *g* at 2–8°C for 15 min. The resulting supernatants were mixed with isopropanol (0.5 mL/1 mL Trizol reagent), incubated at 15–30°C for 10 min, and centrifuged at 12,000× *g* for 10 min at 2–8°C to collect RNA pellets. After twice washing with 75% ethanol, the resulting RNA pellets were dried and resuspended in water or an appropriate buffer.

### Reverse transcriptase-PCR analysis

First strand cDNAs were synthesized at 42°C for 1 h in a 20 µL reaction that contains 2.0 µg of total RNAs, 4.0 µL of 5× reaction buffer, 1.0 µL of oligo d(T)_15_ (50 mmol/L), 2.0 µL dNTP mix (10 mM each), 1.0 µL Ribonuclease Inhibitor (40 U, TAKARA, Japan), 1 µL AMV reverse transcriptase (5 U, TAKARA, Japan). 0.5 µL of the reaction product was used for subsequent PCR amplification of gene-specific cDNA fragments in a 50 mL reaction containing 40 µL of RNase-free H_2_O, 5 µL of 10× PCR buffer, 1 µL dNTP mix (10 mM each), 1 µL of forward primer (10 µmol/L), 1 µL of reverse primer (10 µmol/L), and 0.4 µL of DNA polymerase (2.5 U/µL). The gene-specific primer pairs used for the RT-PCR reactions were: gaagaagaagatgtcttgctg and acagtagcagcatccatacg for *OsMT2b*; gtgcttgtgtgtcattctaccc and ggacatggtggttcagttgaaccc for *OsSRT1*; tgggtctccatatatgttcac and ggactcattccgcgacttac for *OsPDK1*; gccaggctctgggtcag and cgggtcgcgccccacg for *OsPDK2*.

### RNA blot analysis

For RNA blot hybridization, equal amounts (∼20–30 µg) of total RNAs were separated on 1.2% denaturing agarose gels containing 12.5% formaldehyde and transferred on to a Hybond-N nylon membrane (Amersham Biosciences). The hybridization probes were amplified by gene-specific primers used for RT-PCR analysis and were labelled using an [α-^32^P]-dCTP random prime-labelling system. Hybridization was performed at 42°C following a previously described procedure [40]. After hybridization, the membrane was washed twice with 2× SSC containing 0.1% SDS (w/v) and twice with 0.1× SSC containing 0.1% SDS (w/v) at 50°C, and the hybridization signals were visualized by Molecular Imager PharosFX Plus System (Bio-Rad).

### Immunoblot Analysis

Tissues were collected from the transgenic and wild type plants, and total proteins were extracted as described [41]. The protein extracts (100 µg per lane) were separated by SDS-polyacrylamide gel electrophoresis and transferred to Pure Nitrocellulose Blotting Membrane (Pall Corporation) using the wet transfer apparatus. The membranes were incubated in blocking buffer (5% [w/v] skimmed milk powder, 0.05% [v/v] Tween 20, 20 mM Tris-HCl, and 500 mM NaCl, pH 7.5) for 1 h, washed 5 times (5 min each) with TBST (0.05% [v/v] Tween 20, 20 mM Tris-HCl, and 500 mM NaCl, pH 7.5), and incubated with the primary antiserum (1∶500 dilution) for 2 h at room temperature. After 5 rinses (5 min each) with TBST, the membranes were incubated with the secondary antibody (alkaline phosphatase-conjugated goat anti-rabbit IgG [ALP], 1∶10000 dilution; Kirkegaard and Perry Laboratories) for 1.5 h at room temperature, washed 5 times (5 min each) with TBST, and subsequently incubated in the substrate buffer (0.33 mg/mL nitroblue tetrazolium [Sigma-Aldrich], 0.165 mg/mL BCIP [Bio-Basic], 0.1 M Tris, 0.1 M NaCl, and 5 mM MgCl_2_, pH 9.5) for several minutes in the dark, and the chemiluminescent signals were subsequently detected by autoradiography film.

## Supporting Information

Text S1 Construction of RNAi/antisense transgenes.
(DOC)
