# Correction: Expression of RNA-Interference/Antisense Transgenes by the Cognate Promoters of Target Genes Is a Better Gene-Silencing Strategy to Study Gene Functions in Rice

**DOI:** 10.1371/annotation/6818176e-4f00-4229-a133-a83d0c3b11da

**Published:** 2011-07-21

**Authors:** Jing Li, Dagang Jiang, Hai Zhou, Feng Li, Jiawei Yang, Laifa Hong, Xiao Fu, Zhibin Li, Zhenlan Liu, Jianming Li, Chuxiong Zhuang

The third funding source, National Natural Science Foundation of China, grant is incorrect. The correct grant information is: "the National Basic Research Program of China (2007CB108705, http://www.973.gov.cn/English/Index.aspx)" 

