# Supplementary material for: Expression of RNA-Interference/Antisense Transgenes by the Cognate Promoters of Target Genes Is a Better Gene-Silencing Strategy to Study Gene Functions in Rice
Source: PLoS One. 2011 Mar 3;6(3):e17444. doi: 10.1371/journal.pone.0017444 (PMC3048295; doi:10.1371/journal.pone.0017444)
Supplement: Text S1 — Construction of RNAi/antisense transgenes. (DOC) [file pone.0017444.s001.doc]

**SUPPORTING INFORMATION**

## Text S1

## Construction of RNAi/antisense transgenes

**1. Generation of the *pOsMT2b::OsMT2b-*RNAi transgene**

The promoter of *OsMT2b* was amplified by PCR from Zhonghua11 genomic DNA using primer pair P-MT-F and P-MT-R (Table 1). The resulting 1939bp DNA fragment (corresponding to the DNA fragment of -1987 to –49bp [AC079356: 90035-91973] upstream of the predicted translational start codon ATG of the annotated *OsMT2b* cDNA [Os05g0111300: NM_001060982]) was doubly digested with the restriction enzymes *Hin*d III and EcoR V and cloned into the *Hin*d III/*Eco*R V-digested pBluescriptII plasmid vector. The plasmid carrying the *OsMT2b* promoter was digested with *Eco*R V and *Eco*R I and ligated to an *Eco*R V/*Eco*R I-digested PCR fragment (corresponding to the intron 2 plus exon 3 of *OsMT2b* [AC079356: 92251-92789bp]) that was amplified from Zhonghua 11 genomic DNA using primer pair R-MT-1 and R-MT-3 (Table 1).The exon 3 of *OsMT2b* (NM_001060982: 221-644bp) was PCR amplified from the 1st-strand cDNAs derived from total RNAs of Zhonghua 11 using the primer pair R-MT-1 and R-MT-2 (Table 2), digested with *Spe* I and *Eco*R I, and subsequently in the reverse orientation cloned into the *Eco*R I/ *Spe* I digested pBluescriptII plasmid carrying the *OsMT2b* promoter and the intron 2 plus exon 3. After sequencing to ensure no PCR error, the *OsMT2* DNA fragment was subsequently removed from the pBluescriptII vector and cloned into the RNAi binary vector pCAMBIA1380 at its multiple cloning site. The resulting vector was digested with *Hin*d III and *Spe* I and then subcloned to the same sites of the pCAMBIA1380 binary vector (Figure 1A).

**2. Generation of the *pOsSRT1::OsSRT1-*RNAi/antisense transgenes**

The promoter of *OsSRT1* (corresponding to the DNA fragment of –1978 to –49bp upstream of the predicted translational start codon of the *OsSRT1* cDNA [Os04g0271000: NM_001058878] was PCR amplified from the Zhonghua11 genomic DNA using the primer pair P-SRT-F and P-SRT-R (Table 1). The resulting 1930bp DNA fragment was digested with *Eco*R I and *Kpn* I and was subsequently cloned into the *Eco*R I/*Kpn* I-digested pUC18 vector. After double digestion with *Bam*H I and *Sal* I, another *OsSRT1* DNA fragment (corresponding to the intron 14 and exon 15 of the *OsSRT1* gene [AL663014: 130023-131512bp]) was amplified from Zhonghua 11 genomic DNA using the primer pair R-SRT-1 and R-SRT-2 (Table 1) and.cloned into *Bam*H I/*Sal* I-digested pUC18 vector carrying the *OsSRT1* promoter. A 564bp-cDNA fragment corresponding to the exon 15 of the *OsSRT1* gene (NM_001058878: 1206-1769bp) was PCR amplified from the 1st-strand cDNAs derived from total RNAs of Zhonghua 11 using primer pair R-SRT-1 and R-SRT-3. The exon 15 fragment was in the reverse orientation cloned into the *Sal* I/*Hin*d III-cut pUC18 plasmid DNA carrying the *OsSRT1* promoter+ intron 14 and exon 15 to construct the RNAi vector. The promoter-only and promoter + exon 15-carrying pUC18 plasmids were digested with *Sal* I and *Hin*d III and used to clone the antisense vector. After DNA sequencing to ensure no PCR mistake, the *OsSRT1* fragments were cleaved from the pUC18 vector and cloned into the pCAMBIA1380 binary vector using *Eco*R I and *Hin*d III (Figure 1B and 1C).

**3. Generation of *pOsPDK/pUbi::OsPDK*-antisense transgenes**

The *OsPDK1* promoter was PCR amplified from Zhonghua11 genomic DNA using the primer pair P-PDK1-F/R (Table 2). The amplified 1678bp- DNA fragment (covering the region of -1701 to -24bp upstream of the annotated translational start codon of the *OsPDK1* cDNA [Os03g0370000: NM_001056731; AC082644: 112000-113877bp] was digested with *Eco*R I and *Xba* I and ligated into the *Eco*R I/*Xba* I-digested pUC18 vector. The resulting pUC18 plasmid was subsequently cut by *Hin*d III and *Sal* I and used to ligate in the reverse orientation with the *Sal* I/*Hin*d III-digested cDNA fragment PCR amplified from the Zhonghua 11 1st cDNAs using the primer pair S-PDK1-F and S-PDK1-R (Table 1).

A 1913bp DNA fragment (corresponding to the region of –2006 to –94bp upstream of the predicted translational start codon of the *OsPDK2* genomic sequence [Os07g0637300; AP003749: 87838-85738bp] was PCR amplified from Zhonghua11 genomic DNA using the primer pair P-PDK2-F and P-PDK2-R (Table 1). This PCR fragment was digested with *Eco*R I and *Kpn* I and was subsequently cloned into the *Eco*R I/*Kpn* I-digested pUC18 plasmid DNA. The resulting pUC18 plasmid was subsequently cut by *Hin*d III and *Sal* I and used to ligate in the reverse orientation with the *Sal* I/*Hin*d III-digested cDNA fragment PCR amplified from Zhonghua 11 cDNA using primer the pair S-PDK2-F and S-PDK2-R (Table 1).

After DNA sequencing to ensure no PCR error, both *OsPDK1* and *OsPDK2* fragments were cleaved from the pUC18 vector and subsequently cloned into the pCAMBIA1380 binary vector between *Eco*R I and *Hin*d III sites to generate *pOsPDK1::OsPDK1*-antisense and *pOsPDK2::OsPDK2*-antisense constructs (Figure 1D and 1E). The construction procedure for generating *pUbi::OsPDK1/2*-antisense transgenes was the same as described above except that the *Ubi* promoter was amplified from a constructed plasmid carrying the maize Ubi promoter using the primer pair P-Ubi-F and P-Ubi-R (Table 1, Figure 1F and 1G).
